# Inhibition of dicer activity in lepidopteran and dipteran cells by baculovirus-mediated expression of Flock House virus B2

**DOI:** 10.1038/s41598-019-50851-4

**Published:** 2019-10-10

**Authors:** Jeffrey J. Hodgson, Luke W. Wenger, Rollie J. Clem, A. Lorena Passarelli

**Affiliations:** 1 Kansas State University, Division of Biology, Manhattan, KS 66506 USA; 2 Present Address: Boyce Thompson Institute, 533 Tower Rd., Ithaca, NY 14853 USA; 3 Present Address: University of Kansas Medical Center, Department of Anatomy and Cell Biology 3901 Rainbow Boulevard, Mailstop 3038., 66160 Kansas City, USA

**Keywords:** Virus-host interactions, RNAi

## Abstract

Prior studies have suggested that insect DNA viruses are negatively affected by dicer-2-mediated RNA interference (RNAi). To examine this further, we utilized an *in vitro* assay to measure dicer activity in lepidopteran and dipteran cells, combined with baculoviruses expressing the RNAi suppressor B2 from Flock House virus or *Aedes aegypti* dicer-2 (Aedicer-2) using a constitutive heat shock promoter. Addition of cell lysates containing baculovirus-expressed B2 to lysates from dipteran (S2, Aag2) or lepidopteran (Sf9) cells inhibited endogenous dicer activity in a dose-dependent manner, while expression of Aedicer-2 restored siRNA production in *Ae*. *albopictus* C6/36 cells, which are dicer-2 defective. However, B2 expression from the constitutive heat shock promoter had no impact on baculovirus replication or virulence in cell lines or larvae that were either highly permissive (*Trichoplusia ni*) or less susceptible (*Spodoptera frugiperda*) to infection. We determined that this constitutive level of B2 expression had little to no ability to suppress dicer activity in cell lysates, but higher expression of B2, following heat shock treatment, inhibited dicer activity in all cells tested. Thus, we cannot rule out the possibility that optimized expression of B2 or other RNAi suppressors may increase baculovirus replication and expression of heterologous proteins by baculoviruses.

## Introduction

The small interfering RNA (siRNA) pathway is an important antiviral defense mechanism in plants and arthropods, which lack antibody-based immune systems. Dicer (RNase III endonuclease) enzymes, an important component of the siRNA pathway, bind and digest (or dice) long double-stranded RNAs (dsRNAs) into 21-bp siRNAs. The resulting siRNAs direct an RNA-induced silencing complex (RISC) to scavenge and enable Argonaute-mediated enzymatic degradation (slicing) of RNA in a sequence-specific manner. Since RNA viruses produce dsRNA replication intermediates and genomic RNAs with structural elements^1^ that elicit host RNA interference (RNAi) responses, it is not surprising that many RNA viruses have evolved methods to directly antagonize the RNAi pathway. Several plant and insect RNA viruses encode viral suppressors of RNAi (VSRs), that (1) bind to long dsRNA or siRNAs^2^ to avoid cellular dsRNA detection and dicing by dicer-2, or (2) interfere with RISC complex formation and Argonaute-2 slicing activity^3^.

The Flock House virus (FHV) B2 protein^4^, like most VSRs, blocks the initial steps of the siRNA pathway by binding and sequestering long dsRNA or siRNAs produced by dicer-2 activity. Other VSRs such as cricket paralysis virus (CrPV) 1 A^3^ or Nora virus VP1^5^ interfere with downstream siRNA-directed slicing by Argonaute-2 (AGO-2). Individual VSRs may also have multiple functions that modulate RNAi, such as CrPV 1 A, which not only interferes with AGO-2 functions in the siRNA^3^ and miRNA^6^ pathways, but also binds AGO-2 and recruits the host Cul2-Rbx1-EloBC ubiquitin ligase complex, resulting in AGO-2 degradation^7^. Although DNA viruses do not generate obligatory dsRNA during genome replication, they often produce complementary overlapping mRNAs through bi-directional transcription, thereby generating dsRNA that promotes dicer-2-mediated RNAi^8^. Indeed, prior studies suggest that the dicer-2-dependent RNAi pathway is induced^9^ by iridovirus infection and can affect iridovirus replication during infection of *Drosophila melanogaster*^10,11^. Inhibiting RNAi was also found to enhance baculovirus-mediated transgene expression^12^ or baculovirus infection of permissive^8^ and semi-permissive lepidopteran hosts^13,14^. However, the effects of RNAi on the levels of DNA virus replication in insects are relatively modest when compared to the effects on RNA viruses. In addition to *dicer-2*, lepidopteran insects also encode and express orthologues of *dicer-1*^15,16^, an RNase III endonuclease implicated in endogenous pre-microRNA processing^17–19^. Transient VSR expression in cells may also impact microRNA processing, but, herein, we only address the impact of the B2 VSR on dicer-2 activities.

Baculoviruses such as Autographa californica multiple nucleopolyhedrovirus (AcMNPV) are large DNA-containing viruses that are important pathogens of insects, mainly lepidopterans. Baculoviruses have also been developed for many biotechnological applications such as expression of therapeutic proteins, production of virus-like particles as vaccine candidates, and generation of adeno-associated viral vectors for use in gene therapy. RNAi knockdown in lepidopteran systems has been reported to have variable efficiencies^20^; but, if improved, RNAi of host antiviral genes could potentially augment the efficiency of heterologous protein expression. The ability of lepidopteran dicer-2 to digest long dsRNAs into siRNAs has been indirectly verified through deep sequencing of RNA extracted from virus-infected cells^8,11^. However, inhibition of lepidopteran dicer-2 dicing activity has not been extensively explored by direct methods. Understanding the mechanism of dsRNA processing could impact the use of lepidopteran cells and their associated baculoviruses in biotechnology applications by developing methods to suppress dicing activity in lepidopteran cells.

Here, we compared the endogenous dsRNA dicing activities of lepidopteran and dipteran dicer-2 enzymes, and the effect of expressing B2 on dicing in lepidopteran and dipteran cells. We also examined the effect of B2 expression on AcMNPV replication in cell lines and insect larvae. Most prior studies of baculoviruses expressing heterologous VSRs utilized the baculovirus very late polyhedrin (*polh)* promoter, which enables very robust VSR expression. However, using the *polh* promoter has two distinct disadvantages: 1) *polh* expression occurs only in cells that are permissive for baculovirus expression, and 2) *polh* expression begins very late in the baculovirus replication cycle^21^. Here, we expressed B2 from a constitutive *Drosophila hsp70* (HS) promoter^22^. Constitutive B2 expression during the initial phase of baculovirus infection could impact viral early gene expression and thereby modulate the course of infection, and also allows for baculovirus-mediated B2 expression in dipteran cells that do not support baculovirus replication or very late gene expression. Finally, we generated a baculovirus that expressed the *Aedes aegypti dicer-2* (Aedicer-2) (also from the constitutive HS promoter) and assessed the effects of expressing Aedicer-2 or B2 individually or together in permissive lepidopteran or non-permissive dipteran cells.

## Materials and Methods

### Cell culture

*Spodoptera frugiperda*-derived Sf9 cells, a clonal derivative of IPLB-Sf21-AE^23^ were purchased from ATCC, and *Trichoplusia ni*-derived TN-368 cells^24^ were cultured in TC-100 medium with 0.26% tryptose broth (Invitrogen) supplemented with streptomycin sulfate (200 μg/ml), penicillin G (60 μg/ml), and amphotericin B (0.5 μg/ml)]. *Drosophila*-derived S2 and *Ae*. *aegypti-*derived Aag2 cells were cultured in Schneider’s insect medium (Invitrogen). *Ae*. *albopictus*-derived C6/36 cells were cultured in Leibovitz-15 medium (Invitrogen). Media used for all lines was supplemented with 10% heat-inactivated fetal bovine serum (Atlanta Biologicals). Cells were grown at 27 °C.

### Viruses

To construct AcB2, the FHV B2 open reading frame was PCR-amplified with oligonucleotides FHV B2 F (5′-CCTAAGGATGCCAAGCAAACTCGCGCTAATCCAGGAACTTC-3′) and FHV B2 + HA R (5′-CCTAAGGCTAGGCGTAATCTGGGACGTCGTATGGGTACAGTTTTGCGGGTGGGGGGTCAC-3′) and cloned into a plasmid under control of the constitutive *Drosophila* HS promoter sequence^25^ to generate pHSP70-B2. The hsp70-B2-HA cassette was then PCR-amplified with oligonucleotides HS promo-insert-polyA F with an EcoRI restriction site (5′-ACGTACGTACGTGAATTCGGATCCTTAAATTGTATCCTATATTAAAACAGAAGAAAGT-3′) and HS promo-insert-polyA R with a StuI restriction site (5′-ACGTACGTACGTAGGCCTCGAAAATCGGGCTAGATTTAAC-3′) and cloned into the EcoRI and StuI sites of a modified FastBac transposition vector (pFB-PG-pA)^26^.

To generate the AcDCR2 baculovirus expressing dicer-2, the *Ae*. *aegypti dicer-2* open reading frame was PCR-amplified and cloned under control of the HS promoter in the pFB-**Δ**HIS/TEV vector, a pFastBac HTA vector that was modified by deleting the His tag and TEV coding sequences^27^. First, the HS promoter was obtained from pHSP70-B2 by digesting with EcoRI and SacI and inserted downstream of the *polh* promoter in pFB-**Δ**HIS/TEV to produce pFB-PH/HSP70. The DCR2 open reading frame was PCR-amplified using oligo-dT reverse transcribed RNA from Aag2 cells and primers 5′-AAGAGCTCAATATG*CATCACCACCACCATCAT*GGCGGAGGTGATATGATTATGCCACAGC-3′ with a 6X his tag (in italics) and 5′-AATTCTAGAAACATTACTTAGCACTGCGG-3′ and cloned into pFB-PH/hsp70 downstream of the *polh* and *hsp70* promoters using SacI and XbaI (underlined in the oligos) and the corresponding AcDCR2 virus was generated using standard methods described elsewhere^28^. The control virus (AcWT) consisted of the same bacmid virus backbone as that of AcB2 and AcDCR2 but contained the empty pFB-PG-pA vector that was transposed into the bacmid *polh* locus.

For cell infections and transductions, viruses were diluted in TC-100 medium and incubated with cell monolayers for 1 h at room temperature with gentle rocking. Transduction of dipteran cells was carried out using an amount of infectious virus equivalent to 2 PFU/cell (1 PFU/cell for each virus in co-infection studies) as assessed in Sf9 cells. The time when the viral inoculum was removed from cells and replaced with fresh medium was considered 0 h post inoculation or infection. Independent budded virus growth kinetic assays used separate virus stock preparations and were analyzed after three replicate infections. Virus inocula for experiments with lepidopteran cells were titrated in Sf9 or TN-368 cells, as appropriate. Virus concentrations to determine temporal budded virus production kinetics in Sf9 and TN-368 cells were determined in Sf9 cells by end-point dilution^28^.

### Insect studies

Viral occlusion bodies (OBs) from AcB2 and the control parental bacmid AcWT were used for insect dose-response and survival assays. OBs were isolated from infected insects by injecting 4th and 5th instar *T*. *ni* larvae (Benzon Research, PA) with about 1 × 10^4^ TCID_50_ units of the respective budded viruses produced in Sf9 cells. OBs were purified^28^, quantified using a hemocytometer, diluted in sterile water, and added to molten (50 °C) *T*. *ni* insect diet (Southland Products, AR). Neonate *T*.* ni* larvae were placed on OB-contaminated diet within three hours after emerging from eggs and incubated thereafter at 27 °C with a 12/12 h light/dark cycle. Insects were inspected every 8 h for mortality, which was noted by their lack of response to prodding with a blunt glass rod. For survival studies, insects were infected with diet containing OBs that caused 100% *T*. *ni* (1.1 × 10^5^ OBs/ml) mortality or 90% *S*. *frugiperda* mortality (2.6 × 10^7^ OBs/ml) in LC_50_ assays. Lethal concentration analysis was performed using the PROBIT regression module of SPSS software (IBM). Survival analysis was performed using the survival analysis module of SPSS software using Kaplan-Meier statistical parameters.

### Immunoblotting and protein detection

Proteins were resolved by SDS-15% PAGE and transferred to PVDF membranes^29^. Equal total cell protein, determined using BCA assays (Pierce), was analyzed when indicated, whereas equivalent lysate volumes (i.e., cell equivalents) were used when assessing temporal protein production. Membranes were blocked with 5% non-fat milk in a Tris-buffered saline/Tween-20 (TBST) solution (50 mM Tris, 150 mM sodium chloride, 0.05% Tween-20, pH 7.5) and then incubated with primary mouse monoclonal antibodies against HA (1:5000 dilution; HA-7 clone, Sigma) or GP64 (1:1000; AcV5 clone, obtained from Gary Blissard, Boyce Thompson Institute), followed by secondary horseradish peroxidase-conjugated goat anti-mouse (1:10,000, BioRad) antibodies in 1% non-fat milk in TBST. Proteins were detected using SuperSignal West Pico chemiluminescent substrate (Pierce) and X-ray film.

### Preparation of radiolabeled dsRNA enzyme substrates

A 550-bp PCR amplicon of the *chloramphenicol acetyltransferase* gene sequence was generated with 5′ and 3′ flanking T7 promoter sequences (lowercase letters) in the oligonucleotides 5′-taatacgactcactatagggTATCCCAATGGCATCGTAAAGAACA-3′ and 5′-taatacgactcactatagggACAAACGGCATGATGAACCTGAAT-3′) and used for T7-directed RNA transcription using the T7-Scribe kit (Cellscript), according to the instructions of the manufacturer, except that one third of the kit-supplied cytosine triphosphate (CTP) in the reaction was omitted and replaced with α-^32^P-labeled CTP (Perkin Elmer). Unincorporated radionucleotides were removed from the reaction mixture using Sephadex resin G-50 spin columns (GE). The 550-bp dsRNA fragments were resolved in and extracted from 1% agarose gels/TAE and purified using direct-spin nebulizing columns (Lambda Biotech), followed by a reaction clean-up and RNA concentration, using a PCR product purification column (Qiagen). The RNA was then eluted in nuclease-free water. Concentration of dsRNA was determined at 260 nm absorbance of a dsRNA dilution (100×) in nuclease-free water using Beer’s law by the relation of µg/ml dsRNA = A260 × 40 µg/ml. The radiolabeled dsRNA was used a substrate for up to one month after being synthesized.

### Dicer-2 enzymatic activity assay

An *in vitro* protocol for assessing dicer-2 activity levels in cellular lysates was adapted from a method developed for measuring dicer-2 activity in S2 cells^30^; the only modification made was that the final reaction volumes were doubled (from 10 μl to 20 μl) without concomitantly scaling the amount (50 μg) of total lysate protein. This was necessary because we tended to obtain lower protein concentrations from lepidopteran cells. Lepidopteran (Sf9, TN-368) or dipteran (Aag2, C6/36, S2) cells were plated in 100 mm^2^ dishes at approximately 70% confluency and infected or transduced, respectively, 24 h prior to lysate collection. A total of two dishes for TN-368 and S2 cells, three for Sf9, Aag2, and mock-treated cells, and six for mock-treated and transduced C6/36 cells were used to produce lysates. About 70% infection or 30–50% transduction efficiencies were achieved for all experiments based on virus-expressed GFP fluorescence at 24 h post-inoculation. Where indicated, cells were heat shocked to boost expression of B2 or Aedicer-2 by floating culture dishes in a water bath (30 min, 40 °C) at 20 h post-inoculation and incubated for 4 h at 27 °C prior to lysate preparation.

To prepare lysates, cells were resuspended by gentle pipetting (or for C6/36, after scraping from dishes with a rubber policeman) and collected by centrifugation (500 × g, 5 min at room temperature). Cell pellets were washed in phosphate-buffered saline (pH 6.2) with gentle vortexing and centrifugation and then resuspended in ice-cold hypotonic solution (10 mM Hepes-KOH, pH 7.0, 2 mM MgCl_2_, 0.1% β-mercaptoethanol, 1X Roche Protease Inhibitor cocktail), supplemented with KCl (60 mM), and collected by centrifugation at 4 °C. After removing traces of wash buffer using a tissue (Kimwipe), washed cell pellets were resuspended in 50 µl of ice-cold hypotonic solution (without KCl) and incubated on ice for 10 min. Lysate supernatants were collected after Dounce homogenization and centrifugation (20 min at 20,000 × g, 4 °C). Lysates were supplemented with 60% sterile glycerol in water to a final glycerol concentration of 10% and stored frozen (−80 °C) in 15–20 µl aliquots. The protein concentration of one aliquot diluted 10X in water was measured by absorbance using a microtitre BCA assay (Pierce) prior to performing dicer assays. Lysate samples were diluted to 5 µg/µl in hypotonic buffer (lacking KCl), supplemented with glycerol to a final concentration of 10%, before carrying out dicer reactions. For negative control dicing reactions that lacked any lysate protein, 10 μl of just the hypotonic buffer (lacking KCl, with 10% glycerol) was used.

Dicer reactions (20 µl) contained 10 µl of 2X reaction buffer/dsRNA substrate master mix with 2X buffer K^30–33^, 2 µM ATP, 16 U ribonuclease inhibitor (24 U for assays with lepidopteran proteins; Fermentas), and 50 nmole of radiolabeled dsRNA. The reactions were mixed by iterative (10X) pipetting up and down and incubated for 2 h at 30 °C in a thermocycler with a heated lid (45 °C). To stop the reactions, 180 µl of freshly prepared 2X PK buffer (1% SDS, 10 mM Tris-HCl, pH 7.0, 1 mM EDTA and 1 mg/ml proteinase K) was added before incubating at 65 °C for 10 min. After proteinase K digestion, the contents of the reaction (200 µl) were transferred to a 1.5 ml microcentrifuge tube containing 600 µl of absolute ethanol, 30 mM sodium acetate (pH 5.2) and 20 µg of glycogen. After brief vortexing, the tubes were stored at −20 °C overnight and later centrifuged at 16,000 × g for 10 min at 4 °C to pellet total RNA (cellular RNA, radiolabeled siRNAs, and undigested substrates). Dried RNA pellets were resuspended in 5 µl 1X loading buffer (47.5% formamide, 9 mM EDTA, 0.0125% SDS, xylene cyanol, and bromophenol blue) by heating at 65 °C for 10 min and then denatured by placing tubes in a 95 °C heating block for 5 min. Tubes were rapidly transferred to ice until samples were loaded on a pre-run (20 min at 200 V) 0.75 mm denaturing (7.5 M urea in 0.5X TBE) 15% acrylamide gel.

Urea-15% PAGE gels in 0.5X TBE were cast according to instructions for the urea-acrylamide/TBE buffer system (Protogel) no more than one day prior to use, using freshly made ammonium persulfate solution. Gels were pre-run at 200 V for 20–30 min before samples were loaded. Immediately before sample loading, individual wells were flushed to remove built-up urea by pipetting several times using a gel-loading tip. A microRNA ladder (New England Biolabs) was loaded (150 ng) in its supplied buffer and electrophoresed along with samples to serve as a size indicator for the small RNAs. Denatured (5 min, 95 °C and a quick ice chill) samples were loaded and run at 120–150 V until the bromophenol blue dye had migrated 8 cm. Gels were stained with SYBR Gold (Molecular Probes) diluted in 0.5X TBE, blotted with a Kimwipe tissue to remove excess buffer, and heat-sealed in a seal-a-meal pouch prior to visualization under UV. Sealed gels were exposed to a phosphor screen for 3–16 h at room temperature. Exposed phosphor screens were scanned with a General Electric Typhoon 9410 Phosphorimager using the optimal signal setting. For each of the independent experiments, each lysate sample was assayed simultaneously in two to four separate reactions, and the resulting products were then simultaneously processed, electrophoresed and quantified relative to the products of the other samples on the same gel. Imagequant (GE) or ImageJ software was used to analyze the data. Background signals from gels were negligible (and therefore not subtracted), and bands were manually selected using the same area for each lane on a gel. Mean peak signal ratios (treated/mock-treated) were calculated from the three independent experiments that each incorporated two to four replicate reactions of each lysate. The raw quantitative data is provided in Supplemental Table S1. One-way (paired) t-tests were used to compare siRNA production between control C6/36 cells, AcDCR2 transduced, and AcDCR2 and AcB2 co-transduced C6/36 cells. Two-way (paired) t-tests were used to assess differences in siRNA production when Sf9, TN-368 or Aag2 cells were inoculated with AcWT, AcB2 or AcDCR2, or those co-inoculated with AcWT and AcDCR2 or AcB2 and AcDCR2, each with and without a heat shock (30 min, 40 °C) at 20 h post virus addition.

## Results

### Baculovirus-mediated B2 expression

**Figure 1:**
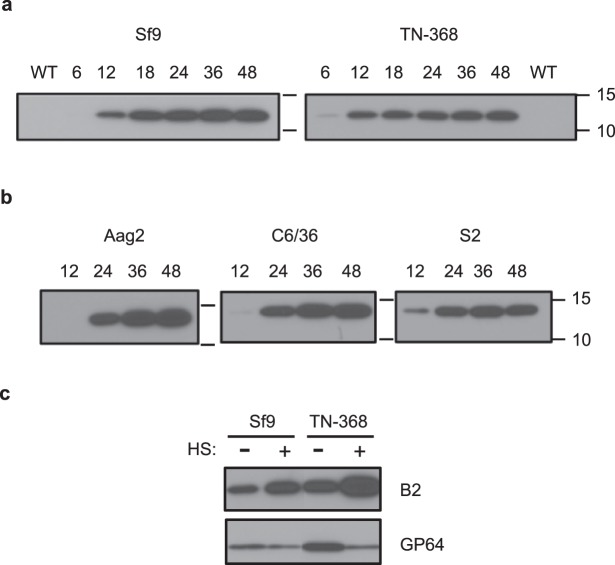
Baculovirus-mediated expression of HA-tagged B2 in lepidopteran and dipteran cells. Lepidopteran Sf9 or TN-368 cells (**a**) or dipteran Aag2, C6/36, or S2 cells (**b**) were infected (**a**) or transduced (**b**) with AcB2 or AcWT (WT) as a control, and cell lysates were collected at the indicated times and immunoblotted to detect B2 (WT was collected at 48 h p.i.). Numbers to the right show the migration of proteins in kilodaltons obtained from protein mass markers. (**c**) Effects of heat shock on protein expression. Immunoblot of B2 in AcB2-infected Sf9 and TN-368 cells at 24 h p.i. with (+) and without (−) heat shock (HS) treatment at 20 h p.i. In A and B, the same volume (i.e., cell equivalents) of lysate was loaded in each lane. In (**c**), equal amounts of total protein (25 μg) were loaded in each lane.

An AcMNPV-based bacmid (AcB2) was used to express a hemagglutinin (HA) epitope-tagged B2 from a *Drosophila* HS promoter in Sf9 or TN-368 cells. HA-tagged B2 was first detected using anti-HA antibodies at 6 or 12 h p.i. in TN-368 or Sf9, respectively, and expression continued through 48 h p.i. (Fig. 1a) in the absence of heat shock induction to boost B2 expression levels. Constitutive HS promoter-expressed B2 was detectable from 6 h p.i. (albeit at relatively low levels), indicating that B2 could impact production of late viral gene products much earlier in the replication cycle than if expressed from the very late *polh* or *p10* promoters. As expected, B2 was not detected in cells infected with the control AcWT virus (Fig. 1a, WT lanes).

B2 expression was also assessed following transduction with AcB2 of three dipteran cell lines: *Drosophila*-derived S2, *Ae*. *aegypti*-derived Aag2, and *Ae*. *albopictus*-derived C6/36. AcMNPV is capable of entering dipteran cells and expressing some of its early genes, but there is no productive infection^31–33^; hence, we refer to this process as transduction rather than infection. Cells were transduced with the equivalent of 2 PFU/cell of AcB2 based on virus titrations in Sf9 cells. AcMNPV was efficient at transducing all three dipteran-derived cell types as indicated by viral *ie-1* promoter-driven GFP reporter fluorescence in ~50% of cells by 24 h post-inoculation (data not shown). B2 was detected in the transduced cells by 12 or 24 h, depending on the cell line, and it accumulated through 48 h post virus addition (Fig. 1b). Overall, the efficient transduction of dipteran cells with AcB2 allowed for robust transient expression of B2.

Although B2 expression from the HS promoter used in these experiments can be easily detected in the absence of heat shock, expression from this promoter can be increased by heat shock treatment^25^. When AcB2-infected lepidopteran cells were heat shocked for 30 min at 40 °C, beginning 4 h prior to harvesting lysates at 24 h p.i., there was an increase in the amount of B2 detected in immunoblots using equivalent amounts of total protein in each sample (Fig. 1c). We also noticed a decrease in GP64, the viral major membrane fusion protein used as a loading control, in the same heat shocked lysate samples. This is likely due to an effect of heat shock on viral protein synthesis, since translation of most cellular proteins is transiently decreased following heat shock treatment, while expression of heat shock proteins is transiently increased^34^. When AcMNPV-infected cells are heat shocked during the early stage of infection, hsp70 protein levels rise and a temporary delay in AcMNPV replication lasting several hours is observed. In contrast, heat shocking cells during the late stage of infection does not stimulate increased hsp70 protein production and, instead, results in rapid death of cells and abortive infection (R. J. Clem and L. K. Miller, unpublished results).

### Rescued dicer-2 activity in C6/36 cells

**Figure 2:**
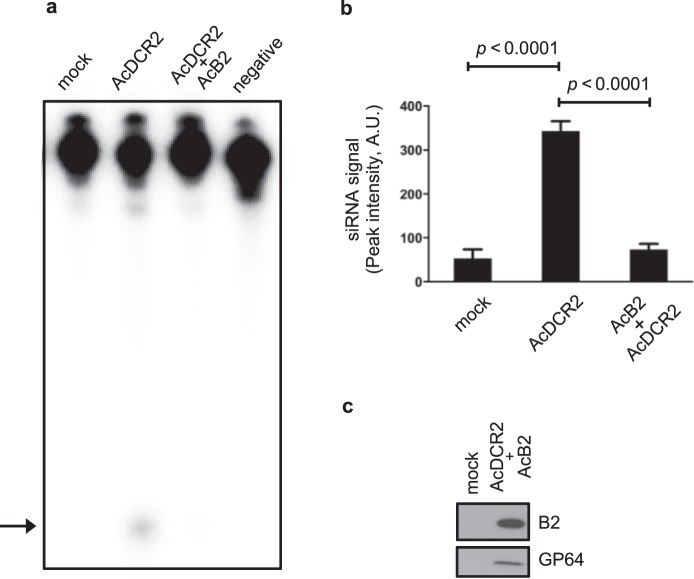
AcDCR2-mediated expression of dicer-2 confers dicer activity in C6/36 cells, as measured by an *in vitro* assay using cell lysates. (**a**) siRNAs were not produced by mock-treated (mock lane) C6/36 cell lysates, but 24 h after transduction with AcDCR2 (and 4 h after heat shock treatment), siRNA production was observed (AcDCR2 lane). Co-inoculation of cells with equivalent volumes of AcDCR2 and AcB2 inocula (AcDCR2 + AcB2 lane) resulted in production of siRNA being substantially suppressed. The negative control lane (negative) consisted of a dsRNA dicing reaction without lysate and was used to control for non-specific dsRNA degradation. Arrow indicates the migration of siRNAs. (**b**) Quantitative production of siRNA in transduced C6/36 cells based on two independent assays. The amount of labeled siRNA was compared between mock-treated, AcDCR2-transduced and AcDCR2/AcB2 co-transduced C6/36 cells. Peak intensity values presented are in arbitrary units (A.U.). Error bars represent standard deviations from the mean. (**C**) AcDCR2/AcB2 co-transduced cells contain detectable B2, unlike mock-treated cells. The baculovirus GP64 protein was detected on the same blot using anti-GP64 antibody. In (**c**), 25 μg of total soluble protein was loaded in each lane.

*Ae*. *albopictus*-derived C6/36 cells are known to lack dicer-2 activity due to a frameshift mutation in *dicer-2*^35,36^. To test the effectiveness of the *Ae*. *aegypti dicer-2* in blocking AcB2-mediated suppression of siRNA production, we engineered an AcMNPV bacmid (AcDCR2) that expresses *Ae*. *aegypti* dicer-2 from the HS promoter. We first transduced *Ae*. *albopictus*-derived C6/36 cells with AcDCR2 (Fig. 2) and tested the ability of lysates from these cells to produce siRNA in an *in vitro* assay using a radiolabeled dsRNA substrate. As expected, lysate from mock-treated C6/36 cells was unable to dice the labeled dsRNA into small RNAs (Fig. 2a, mock lane). In contrast, lysates from AcDCR2-transduced (and heat shocked) C6/36 cells produced detectable levels of labeled small RNAs (Fig. 2a,b). Co-transduction with AcB2 significantly (*p* < 0.0001) inhibited this activity (Fig. 2a,b). B2 expression was verified by immunoblotting (Fig. 2c). The size of these small RNAs was consistent with that of siRNAs (see below). Therefore, we concluded that siRNA production could be rescued in C6/36 cells by expression of *dicer-2* (Fig. 2).

### Endogenous dicer-2 activity in dipteran and lepidopteran cells

**Figure 3:**
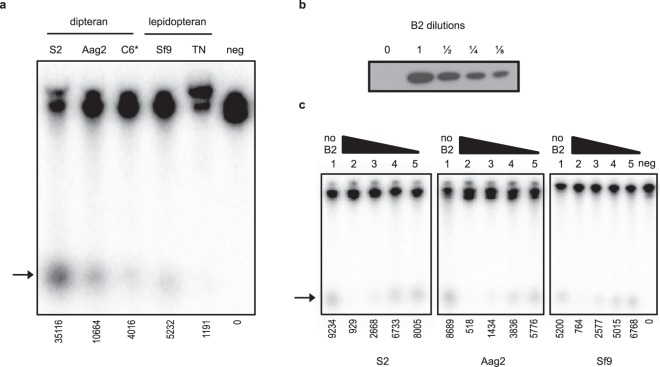
Analysis of endogenous dicer-2 activity in cell lysates using a radiolabeled dsRNA substrate. (**a**) Dipteran and lepidopteran cytosolic proteins were analyzed for siRNA production using a labeled dsRNA and resolved using urea-PAGE. The C6/36 cell lysate (C6*) was derived from cells transduced with the AcDCR2 virus 24 h prior to lysing cells. The numbers listed below each lane refer to the band intensities relative to the negative control lane (neg = 0), as determined using ImageJ. (**b**) Immunoblot of B2 in 2-fold dilutions of lysate used for inhibition assays. (**c**) No B2 (lane 1) or B2-containing C6/36 lysates were added in decreasing concentrations (lanes 2 to 5, noted by the triangle) to cytosolic proteins from S2, Aag2, or Sf9 cells and analyzed for siRNA production. Negative control lanes (neg) in panels (**a**,**c**) contained reactions lacking lysate proteins to control for background dsRNA degradation. Arrows in panels (**a**,**c**) indicate migration of siRNAs.

Previous reports using *in vitro* assays and dipteran cells have shown that dicer-2 can process exogenously supplied radiolabeled dsRNA substrates. However, to our knowledge, similar studies using lepidopteran cells have not been reported. We thus compared the ability of dipteran (S2 and Aag2) and lepidopteran (Sf9 and TN-368) cell lysates to dice radiolabeled dsRNA (Fig. 3). For comparison, we also used lysate from C6/36 cells transduced with AcDCR2 (Fig. 3a, lane C6*). Lysate from each cell line tested was able to produce small RNAs consistent with the size of the small RNAs produced by AcDCR2-transduced C6/36 cells (Fig. 3a). To further characterize the size of these small RNAs, we compared the size of the small RNAs yielded from our dicing assay in S2 lysates to the migration of small RNAs from a commercial microRNA ladder (Supplemental Fig. S1). This analysis determined that the S2 cell-derived small RNAs we detected were approximately 19.5 nt in length, which is close to the reported size (21 nt) of siRNAs^37^. This result, coupled with the observation that these small RNAs are not produced by C6/36 cell lysates (lacking dicer-2), but are produced when dicer-2 is expressed in C6/36 cells, gave us confidence that the small RNAs we observed were indeed siRNAs.

We consistently noted different levels of dicing efficiency from equivalent amounts (50 µg) of total protein from different cell types under the conditions used (Fig. 3a). *Drosophila* S2 cell lysates were the most efficient, while TN-368 were the least. It is possible that S2 cell lysates were more efficient because the assay protocol used was initially optimized for these cells^30^, and we did not attempt to optimize the conditions for the other cell types. Although the siRNA produced from TN-368 cell lysate was harder to detect, quantifiable levels were obtained (Fig. 3a, phosphorimager counts are indicated below each lane). In repeated assays using TN-368 lysates, we often noticed decreased levels of the long dsRNA substrate without a concomitant increase in detectable siRNA, suggesting there may have been non-specific degradation of long dsRNAs in the TN-368 lysates (data not shown). On the other hand, when lysates from the other cell types were used, decreasing amounts of residual long dsRNA substrates were always consistent with higher levels of siRNA.

### Suppression of *in vitro* siRNA production by B2

To assess the ability of B2 to suppress dsRNA dicing in dipteran and lepidopteran cell lysates, we exogenously added 2-fold dilutions of a B2-containing lysate (Fig. 3b) from AcB2-transduced, non-heat shocked C6/36 cells to S2, Aag2 or Sf9 cell lysates. In all cases, undiluted B2 lysate addition (Fig. 3c, lanes 2) significantly inhibited siRNA production. After dilution of the exogenously supplied B2, siRNA production was gradually restored (Fig. 3c, lanes 3 to 5). This indicated that B2 can inhibit dipteran- and lepidopteran-derived dicer-2 activities *in vitro* in a dose-dependent manner.

**Figure 4:**
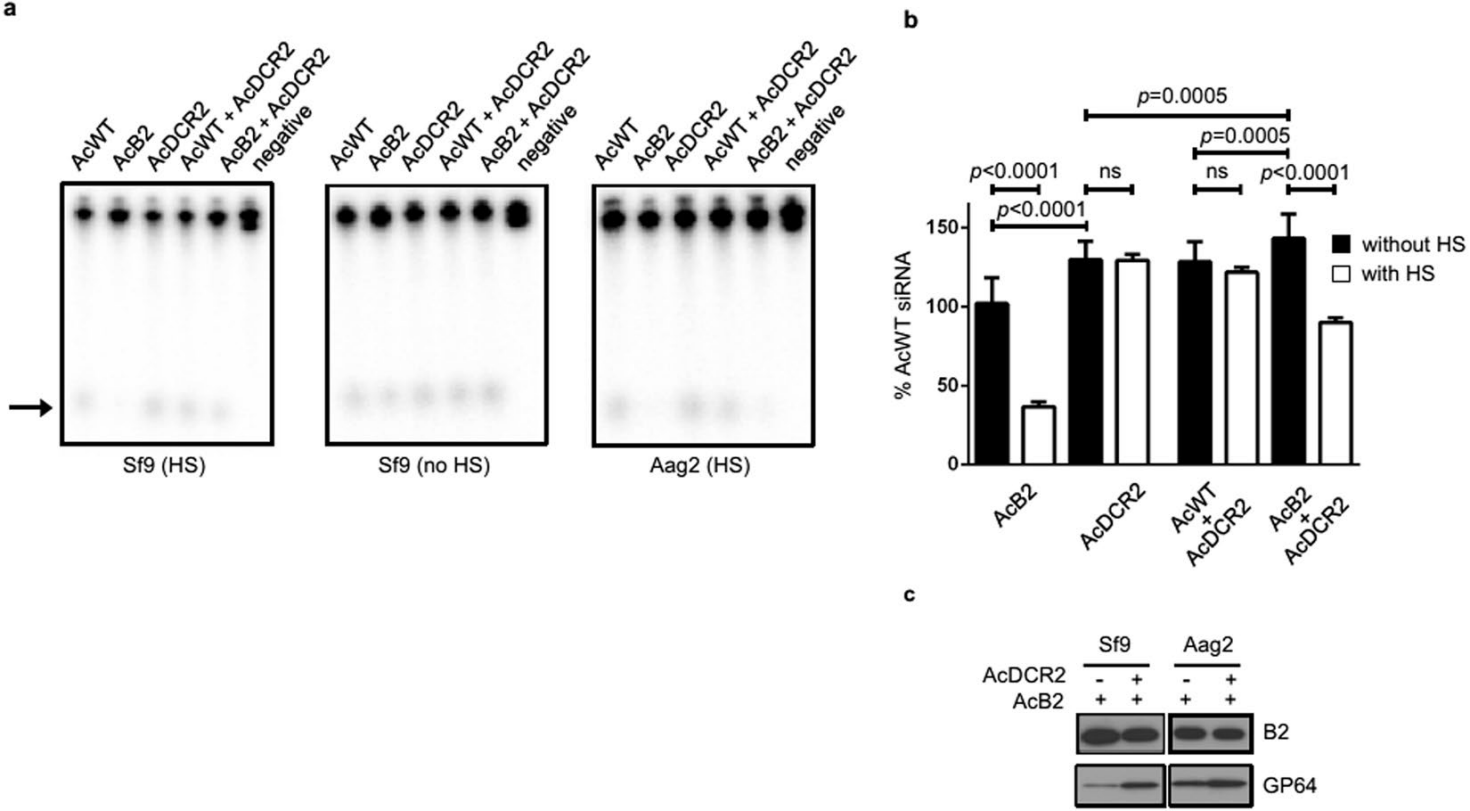
siRNA production in lysates from AcDCR2-transduced or -infected Aag2 or Sf9 cells. (**a**) Lysates from cells transduced/infected with AcWT, AcB2, AcDCR2 or co-transduced/infected with AcWT and AcDCR2 or AcDCR2 and AcB2 were used in *in vitro* dicing assays. The negative control (negative) consisted of a dsRNA dicing reaction without lysate. Arrow indicates the migration of siRNAs. (**b**) Quantification of siRNAs produced in lysates from Sf9 cells infected for 24 h with AcWT, AcB2 or AcDCR2 alone or in co-infections as indicated. Data is expressed as relative to AcWT siRNA, with and without heat shock (HS) treatment at 20 h p.i. Error bars represent standard deviations from the mean. (**c**) B2 in lysates used for dicer-2 assays. Equivalent total soluble lysate protein from AcB2-infected and AcB2/AcDCR2 co-infected (Sf9) or co-transduced (Aag2) cells were immunoblotted with anti-HA antibody to detect B2 or an anti-GP64-specific antibody. The immunoblotted protein samples were derived from heat-shocked samples used for the dicer-2 assays. In (**c**), 25 μg of total soluble protein was loaded in each lane.

We next assessed the effect of increasing the levels of B2 expression (by heat shock induction) on the ability to suppress dicer-2 activity. In Sf9 cells, infection with AcB2 caused decreased siRNA production but only when the cells were heat shocked 4 h prior to harvesting the lysate (Fig. 4a,b). Lysates from AcB2-infected Sf9 or AcB2-transduced Aag2 cells had similar levels of B2 compared to AcB2/AcDCR2 co-infected cell lysates by anti-HA immunoblot analysis (Fig. 4c). We also determined the steady state levels of the baculovirus membrane fusion protein (GP64), and in both cell types we detected slightly increased GP64 when co-inoculated with AcB2 and AcDCR2, consistent with using more input virus to infect the cells. It should be noted that GP64 is expressed during both the early and late stages of baculovirus infection, and thus is expressed in non-permissive Aag2 cells.

### Effects of B2 expression on AcMNPV replication *in vitro* and *in vivo*

**Figure 5:**
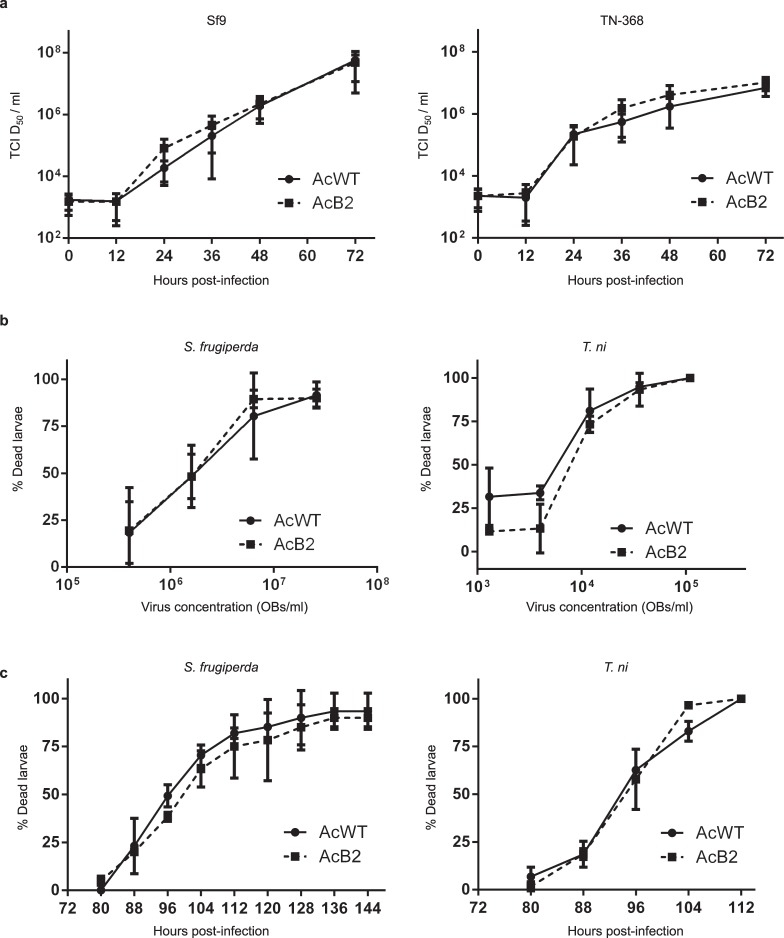
Comparison of AcB2 and AcWT infectious budded virus production in lepidopteran cells and virus lethality in insects. (**a**) Budded virus production. Sf9 and TN-368 cells were infected at an MOI of 0.01 PFU/cell and infectious budded virus production was measured from 0 to 72 h p.i. Data is expressed as the mean budded virus titer (TCID_50_) over time and represents results from three independent experiments. (**b**) Lethal dose analysis. *Spodoptera frugiperda* and *Trichoplusia ni* neonates were infected at various doses (5-fold dilutions) with AcWT or AcB2 occlusion bodies and monitored for death from 0 to 144 h p.i. Error bars represent standard deviations. (**C**) Larval survival analysis. *S*. *frugiperda* and *T*. *ni* neonates were infected with AcWT or AcB2 (2.6 × 10^7^ or 1.1 × 10^5^ OBs/ml from *S*. *frugiperda* or *T*. *ni*, respectively) and insect lethality was monitored every 8 h. The data represent results from two independent experiments that each included 30 insects per group. Error bars indicate the 95% confidence interval limits.

We next assessed whether expression of B2 affected AcMNPV replication in cultured cells or virulence in larvae. Infectious budded virus production in Sf9 and TN-368 cells was determined by end-point dilution titration after infection with AcB2 or AcWT at a low MOI of 0.01 PFU/cell. AcWT and AcB2 exhibited similar infectious budded virus production kinetics in both cell types (Fig. 5a). These data indicate that constitutive HS promoter-driven B2 expression does not appear to affect the amounts of infectious budded virus production. We also compared mean lethal concentration and survival times of neonate *S*. *frugiperda* and *T*. *ni* larvae that were orally infected with OBs derived from AcB2 or AcWT. Again, we did not observe any significant lethal concentration differences between infections with the two viruses in either larval species (Fig. 5b,c, Table 1). However, we observed that AcB2-infected *T*. *ni* larvae took longer to die than AcWT-infected larvae (Table 1). Unfortunately, we were not able to test the effect of increasing B2 expression via heat shock in these virus replication experiments, since heat shock treatment inhibits AcMNPV replication (R. J. Clem and L. K. Miller, unpublished results). In summary, expression of B2 from the HS promoter did not appear to affect the production of infectious budded virus in cultured cells or virus virulence in insects.

**Table 1:** Biological activities of AcWT and AcB2.

			95% Fiducial limits	
		LC_50_ (OBs/ml)	Lower	Upper
*T*. *ni*	AcWT	6680	643	11799
	AcB2	12320	5981	22321
*S*. *frugiperda*	AcWT	1.97 × 10^6^	4.17 × 10^5^	5.42 × 10^6^
	AcB2	1.65 × 10^6^	4.54 × 10^5^	3.91 × 10^6^
			**95% Confidence intervals**	
		**LT**_**50**_^**a**^ **(h)**	**Lower**	**Upper**
*T*. *ni*	AcWT	96	93.59	98.41
	AcB2	104	100.276	107.725
*S*. *frugiperda*	AcWT	104	100.148	107.724
	AcB2	104	100.156	107.844

Notes:

^a^Median survival times in h post-infection when insects were infected with diet containing 1.1 × 10^5^ OBs/ml (for *T*. *ni*) or 2.6 × 10^7^ OBs/ml (for *S*. *frugiperda*) of each virus. Data combines results from two independent assays.

### B2 suppression of endogenous dicer-2 activity

**Figure 6:**
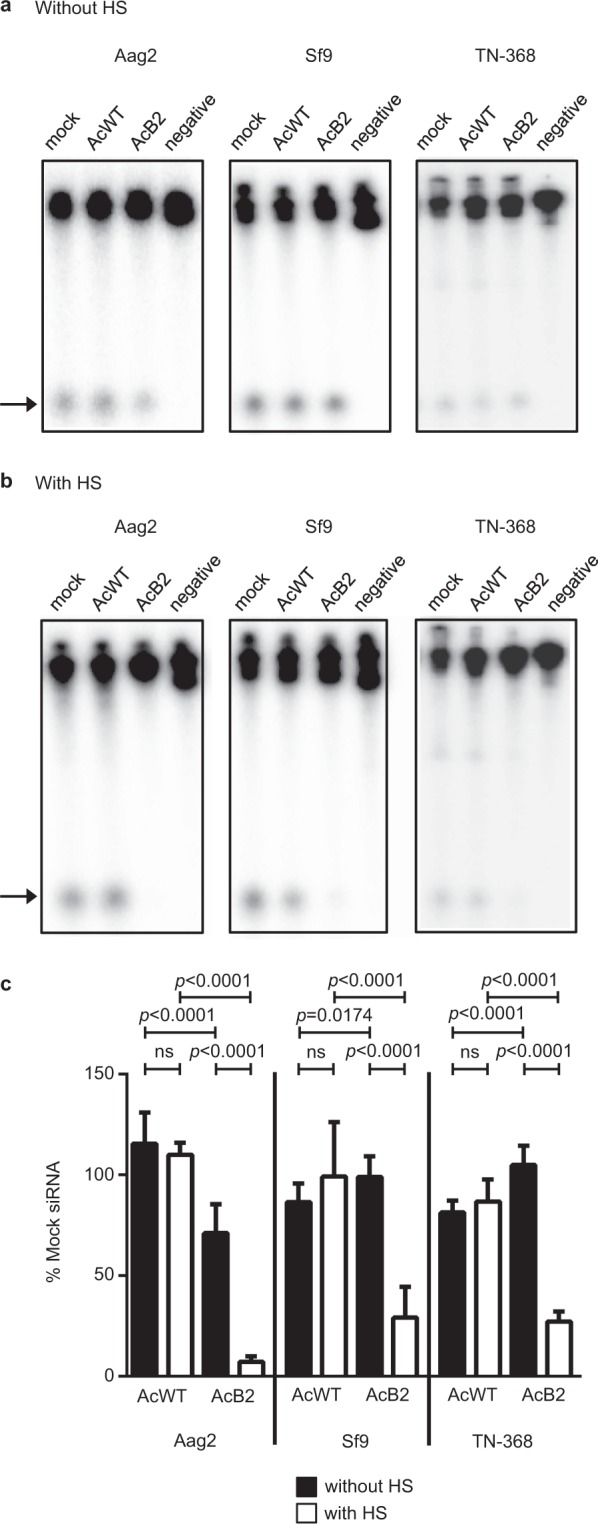
Dicer-2 assays using mock-, AcWT- and AcB2-infected/transduced cells without (**a**) or with (**b**) heat shock (HS) treatment. The negative control (negative) consisted of dsRNA dicing reactions without lysate. (**c**) Quantitation of siRNAs in (**a**,**b**), expressed relative to mock-treated cells. In (**a**,**b**), cell lysates were generated at 24 h post virus addition. The heat shock was administered at 20 h post virus addition in (**b**). Error bars represent standard deviations from the mean. Arrows indicate the migration of siRNAs.

Since our results (Fig. 4) suggested that heat shock might be necessary to obtain sufficient B2 expression to affect dicer-2 activity, we decided to more carefully examine the ability of virus-expressed B2 to inhibit endogenous dicer activity. To do this, we used lysates derived from AcB2-infected or -transduced cells that either did not receive (Fig. 6a) or received (Fig. 6b) heat shock treatment to induce higher B2 expression *in vitro* dicer assays.

When Aag2 cells were transduced with AcB2 in the absence of HS, there was a significant decrease in siRNA production and with heat shock this difference was more pronounced (Fig. 6c). In contrast, in Sf9 and TN-368 cells, we only saw a significant decrease in the amount of siRNA produced when the cells were heat shocked to induce B2 expression (Fig. 6c). Therefore, regardless of whether cells were heat shocked or not, endogenous dicer-2 activities were more effectively suppressed by B2 in AcB2-transduced dipteran Aag2 lysates than in AcB2-infected lepidopteran Sf9 or TN-368 cells.

## Discussion

Dicer-2-dependent antiviral RNAi in insects has been mainly described in relation to regulating genome replication and gene expression of RNA viruses. However, there is also evidence that the dicer-2 siRNA pathway can target dsRNA elements produced during insect DNA virus replication. Prior studies suggest that the dicer-2-dependent RNAi pathway can affect DNA virus replication during replication of an iridovirus in the dipteran *Drosophila*^11^ and during baculovirus infection of permissive^8^ and semi-permissive^14^ lepidopteran cells and insects^13^. Overall, however, the impact of an active RNAi response on insect DNA virus replication appears to be much less than that observed with RNA viruses^38^. Similarly, when tombusvirus P19, a plant virus VSR that binds siRNAs^39^, was expressed in baculoviruses under the control of promoters with different temporal activities (early *ie-2* or very late *p10*), only slightly different levels of budded virus production were observed, although enhanced heterologous gene expression was noted^12^. While there are numerous reported examples of successfully using RNAi in lepidopteran cells, the success of targeted dicer-2 or siRNA-mediated RNAi is more variable in lepidopteran than dipteran systems^20^. Notably, there is little information on the dicing activity of lepidopteran dicer-2 and regulation thereof during baculovirus infection of lepidopteran cells, commonly used for heterologous protein production and other biotechnological applications. A better understanding of lepidopteran host RNAi responses at the molecular and biochemical levels could help improve methods used for the production of proteins, viral-like particles, AaV-based or other viral gene therapy vectors.

In this study, we used an *in vitro* approach to examine the dicing of exogenously added radiolabeled dsRNA targets in protein lysates from uninfected and baculovirus-infected cells. We found that lysates from uninfected lepidopteran cells (Sf9 and TN-368), like dipteran cells (*Drosophila* S2 and *Ae*. *aegypti* Aag2), were capable of processing an exogenous dsRNA substrate into small RNAs that are consistent in size with siRNAs. We verified that lysates from naturally dicer-2-deficient *Ae*. *albopictus* C6/36 cells did not produce these small RNAs, also consistent with the production of the observed small RNAs being dicer-2-dependent. Furthermore, C6/36 cells transduced with a baculovirus expressing *Ae*. *aegypti dicer-2* rescued the production of small RNAs that co-migrated with those derived from the other dipteran and lepidopteran lysates, confirming that the small RNAs we observed were siRNAs.

Virally-derived siRNAs mapping to the genome of the group II alphabaculovirus Helicoverpa armigera single NPV have been described^8^, establishing that lepidopteran dicer-2 can target baculovirus RNAs. Furthermore, transcriptome analysis of AcMNPV-infected *T*. *ni*-derived cells showed diminished *dicer-2*, but not *dicer-1*, transcript levels as early as 1 h p.i.^40^. Reduced *dicer-2* expression due to baculovirus infection is consistent with the finding that replication of the alphanodavirus, a virus naturally present in the *T*. *ni*-derived Hi5 cell line, is stimulated by AcMNPV infection of persistently infected Hi5 cells^41^. However, using an *in vitro* approach, we did not observe any reduction in siRNA production in wild-type AcMNPV-infected cells compared to uninfected controls. Even though AcMNPV infection was previously found to reduce *dicer-2* transcript levels, we used a relatively low multiplicity of infection (MOI = 1.0 PFU/cell) compared to an MOI of 10 PFU/cell used during *dicer-2* expression analysis^40^, so we cannot state with certainty whether *dicer-2* transcript levels were affected in our experiments. Our results do suggest, however, that AcMNPV does not express any VSRs that function upstream of dicer-2. This is in contrast to the large DNA, insect-specific IIV-6 virus that encodes a dsRNA-binding VSR (340 R) that inhibits dicing^42^. The AcMNPV apoptosis inhibitor P35 has been shown to have a distinct role in RNAi suppression by inhibiting the RNAi pathway downstream of dicer-2-mediated dicing^43^.

We showed that baculovirus-expressed B2 was able to inhibit endogenous dicer-2 activity in a dose-dependent manner by adding dilutions of B2-containing lysates to lysates from uninfected lepidopteran and dipteran cells. Lepidopteran and dipteran cells inoculated with a baculovirus constitutively expressing B2 also produced less siRNA compared to control virus-treated cells. However, the level of B2 expression appeared to matter. In lepidopteran cells, B2 expression from the HS promoter was not high enough to inhibit endogenous lepidopteran dicer-2, unless the cells were heat shocked to increase the level of B2 expression. Nonetheless, this demonstrated that B2 produced by baculoviruses in dipteran and (heat-shocked) lepidopteran cells suppressed dicing of exogenous dsRNA substrates. In other studies, suppressing the siRNA pathway reduced the amount of baculovirus-^8,14^ or iridovirus-specific^11^ vsiRNA produced during virus infection. In those studies, small RNA sequencing and Northern blots were used to compare vsiRNA levels, whereas we measured siRNA production from an exogenous radiolabeled non-viral dsRNA by active dicer-2 enzyme in cell extracts. Nevertheless, our results suggest that baculoviruses do not inherently modify host dicer-2 dsRNA dicing. A recently discovered dsRNA-inducible nonspecific (ssRNA, dsRNA, dsDNA) nuclease (up56/REase) encoded by lepidopterans (but not dipterans) was shown to reduce effectiveness of dsRNA-based RNAi knockdown in lepidopteran and dipteran (*Drosophila*) systems^44^. Perhaps the low level of siRNA production and non-specific dsRNA degradation by TN-368 lysates was due to this enzyme. Although *up56/REase* transcription is induced upon dsRNA exposure of cells, it is possible that baculovirus infection also induces *up56/REase*, which resulted in the lower level of dicing observed in our assays.

The ability of B2 to suppress siRNA production in our assays differed between the AcB2-infected lepidopteran and -transduced dipteran cells. At constitutive (non-heat shocked) levels of HS-driven B2 expression, B2 was able to partially inhibit siRNA production in dipteran Aag2, but not in lepidopteran Sf9 or TN-368 lysates; yet, in all of our dicer assays with B2-containing lysates derived from cells that induced higher levels of B2 expression by heat shock, we observed significant reductions in siRNA production. Consistent with the level of B2 expression being an important factor, heat shock treatment resulted in a marginal increase in B2 levels in virus-inoculated dipteran and -infected lepidopteran cells, as measured by immunoblotting. In AcB2-transduced dipteran cells, siRNA production levels were intermediate between that of the AcWT and the heat shocked AcB2-transduced samples. On the other hand, B2 produced in AcB2-transduced C6/36 cells was equally effective at suppressing siRNA production when exogenously added to either uninfected or AcWT-infected lepidopteran cells. Therefore, we conclude that although expressing the FHV B2 VSR from the HS promoter did not affect AcMNPV replication in our experiments, it is capable of inhibiting endogenous dicer-2 activities, but the level of B2 expression is important for function. We were unable to test the effect of increasing B2 expression via heat shock on virus replication, since heat shock treatment affects AcMNPV replication. Thus, further studies to examine production of various heterologous proteins co-expressed with B2 (or other VSRs) in the baculovirus expression system using other promoters are needed.

## Supplementary information

Supplemental Material

## Data Availability

All data generated or analysed during this study are included in this published article (and its Supplementary Information Files).
