# Supplementary material for: Inhibition of dicer activity in lepidopteran and dipteran cells by baculovirus-mediated expression of Flock House virus B2
Source: Sci Rep. 2019 Oct 10;9:14494. doi: 10.1038/s41598-019-50851-4 (PMC6787241; doi:10.1038/s41598-019-50851-4)
Supplement: Supplementary file 1 — Supplemental Material [file 41598_2019_50851_MOESM1_ESM.pdf]

Supplemental material for:

Inhibition of dicer activity in lepidopteran and dipteran cells by baculovirus-mediated expression of Flock House virus B2

Jeffrey J. Hodgson[a,b,*], Luke W. Wenger[b], Rollie J. Clem[a] and A. Lorena Passarelli[a].
[a] Kansas State University, Division of Biology, Manhattan, KS 66506
[b] Current addresses:

JJH: Boyce Thompson Institute, 533 Tower Rd., Ithaca NY 14853; jjh364@cornell.edu

LWW: University of Kansas Medical Center, Department of Anatomy and Cell Biology 3901 Rainbow Boulevard, Mailstop 3038, Kansas City, KS 66160; lwenger3@kumc.edu

*Corresponding author:
JJH: jjh364@cornell.edu

a

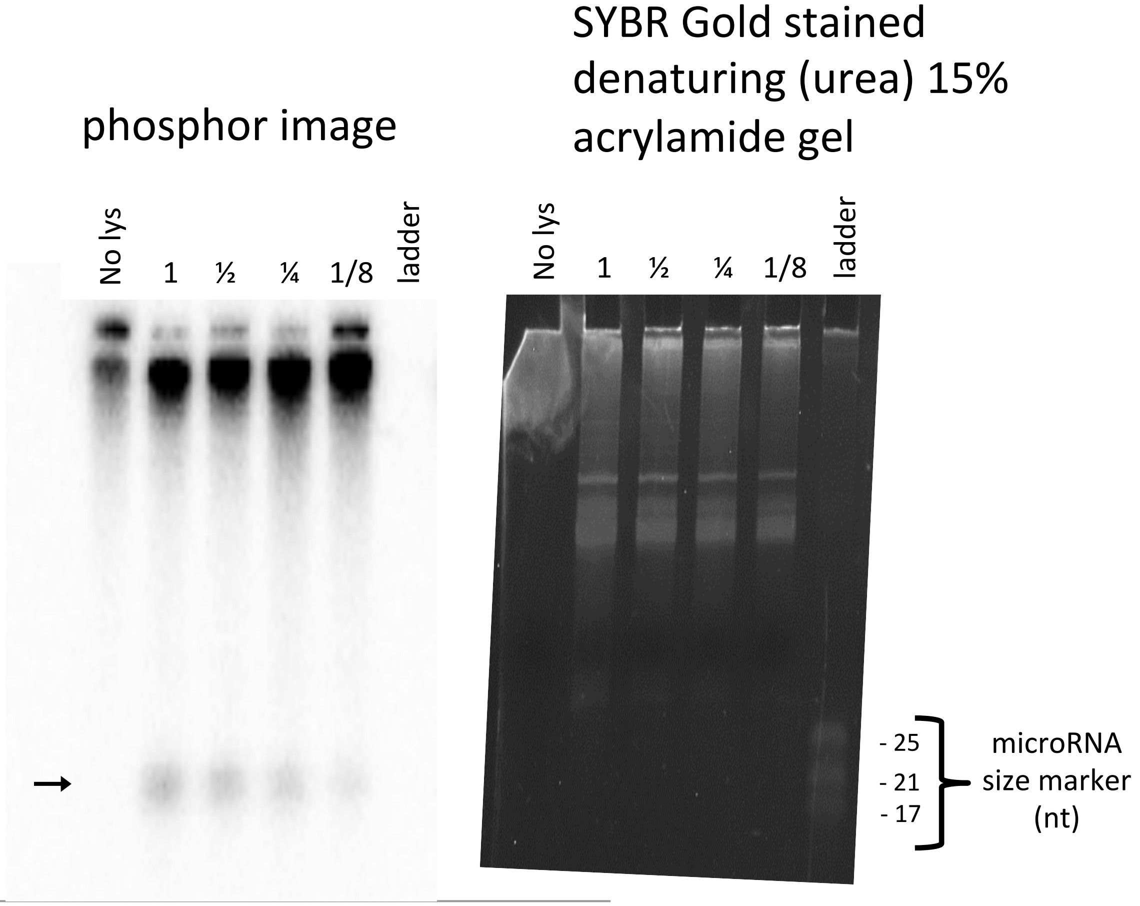

b

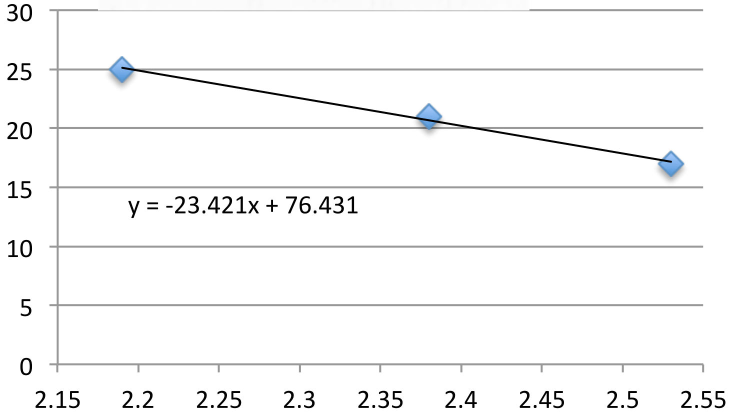

M_r (cm)	size (nt)
y	x
2.19	25
2.38	21
2.53	17

2.43 cm = migration distance of ^{32}P labeled small RNA

interpolated small RNA size = 19.5

Supplemental Figure S1. Characterization of small RNAs produced by in vitro dicing reactions. (A) S2 cell cytoplasmic lysate was isolated as described in the methods. In vitro dicing reactions were carried out using 50 ng (and two fold dilutions thereof; 1 = 50 ng, ½ = 25 ng, ¼ = 12.5 ng, 1/8 = 6.25 ng) total soluble protein in standard dicing assays using 50 nmole of a ^{32}P-labelled dsRNA as described in the methods. RNA was purified from dicing reactions and migrated on denaturing (urea) 15% acrylamide gels as described in the methods alongside a microRNA ladder (NEB) then stained with SYBR Gold and photographed under UV illumination. The wet gel was sealed in plastic and exposed to a phosphor screen. The phosphor image was captured using a phosphorimager (GE). The control reaction contained only lysis buffer and no lysate protein, as described in the methods. Arrow indicates the migration of siRNAs. (B) Linear regression of microRNA ladder bands (25, 21, and 17 nt in length). The migration distance of the SYBR Gold stained microRNA ladder bands in the gel were measured and used to generate the plot shown, from which the equation of the line of best fit was used to interpolate the size of ^{32}P-labelled small RNAs detected on the phosphor screen.

Table S1: Quantification of ^{32}P-labelled siRNAs[1] detected on phosphor screens

Figure 2

C6/36 PLUS HS

	Peak + Background			
**Experiment #**	**Mock**	**AcDCR2**	**AcDCR2/AcB2**	**Area**
1	71.0	335.7	76.7	858
	40.8	346.0	80.5	
	54.6	352.8	73.7	
	38.0	311.6	80.2	
2	49.0	368.2	68.9	1036
	41.9	362.7	68.4	
	60.5	310.1	68.3	
	39.9	357.8	88.8	

Figure 4

Sf9 NO HS

	Peak + Background					
**Experiment #**	**AcWT**	**AcB2**	**AcDCR2**	**AcWT/AcDCR2**	**AcB2/AcDCR2**	**Area**
1	186.5	210.5	217.8	219.0	252.9	910
	163.1	195.2	225.3	231.6	255.8	
	142.8	197.5	211.1	225.7	242.8	
	158.3	182.8	206.5	221.8	244.1	
2	192.3	187.6	233.8	229.5	279.8	1204
	195.0	173.3	241.2	221.4	247.7	
	169.5	153.9	234.4	209.3	261.3	
	176.5	175.7	254.1	227.3	281.0	
3	356.5	304.2	401.6	434.6	469.2	1064
	282.7	249.6	394.1	343.6	380.6	
	320.0	279.8	386.1	407.1	413.5	
	296.9	295.7	365.8	380.5	363.7	

Sf9 PLUS HS

	Peak + Background					
**Experiment #**	**AcWT**	**AcB2**	**AcDCR2**	**AcWT/AcDCR2**	**AcB2/AcDCR2**	**Area**
1	365.6	145.0	445.3	438.7	318.2	812
	342.3	134.7	443.2	430.6	319.9	
2	316.1	106.8	415.1	375.9	282.9	1260
	342.3	134.7	443.2	430.6	319.9	
3	316.1	106.8	415.1	375.9	282.9	1540
	348.9	118.0	462.5	422.4	303.2	

Figure 6

Aag2 NO HS

	Peak + Background			
**Experiment #**	**Mock**	**AcWT**	**AcB2**	**Area**
1	254.7	310.5	189.4	980
	255.0	287.6	178.0	
	187.4	283.4	194.3	
2	271.4	285.8	187.4	1148
	314.4	352.0	221.5	
	326.4	341.9	195.1	
3	324.8	346.1	194.5	1064
	300.0	331.3	188.9	

Aag2 PLUS HS

	Peak + Background			
**Experiment #**	**Mock**	**AcWT**	**AcB2**	**Area**
1	1423.2	1619.8	107.8	1148
	1448.5	1491.3	53.7	
	1128.5	1226.8	120.5	
2	1281.3	1502.6	48.6	1400
	1253.5	1281.3	107.9	
	1343.2	1420.2	73.1	
3	1400.0	1571.9	89.3	1512
	1128.5	1226.8	120.5	
	1240.6	1470.9	117.2	

Sf9 NO HS

	Peak + Background			
**Experiment #**	**Mock**	**AcWT**	**AcB2**	**Area**
1	215.6	183.1	226.4	1110
	194.1	153.3	216.4	
	190.8	158.0	210.9	
	202.6	157.2	230.3	
2	239.8	191.9	240.6	1064
	217.5	169.4	196.6	
	202.4	158.7	188.6	
	209.9	176.1	179.0	
3	1443.8	1465.3	1522.0	1148
	1454.6	1443.5	1421.8	
	1442.1	1372.0	1249.9	
	1513.1	1484.8	1339.6	

Sf9 PLUS HS

	Peak + Background			
**Experiment #**	**Mock**	**AcWT**	**AcB2**	**Area**
1	241.2	233.5	63.3	1170
	225.4	216.8	55.1	
	234.0	214.7	57.8	
	236.3	216.5	56.9	
2	1438.9	1064.7	200.7	1290
	1381.1	1011.8	226.8	
	1286.6	900.9	179.2	
	1397.8	905.3	164.0	
3	1286.8	1792.0	692.7	1232
	1301.2	1715.8	633.3	
	1310.4	1655.0	607.0	

TN-368 NO HS

	Peak + Background			
**Experiment #**	**Mock**	**AcWT**	**AcB2**	**Area**
1	189.4	149.1	218.6	1204
	168.0	127.4	177.3	
	168.8	139.3	186.0	
	197.3	144.9	207.0	
2	116.6	97.7	125.2	1008
	112.0	94.5	115.5	
	107.8	90.2	116.3	
	115.5	102.9	113.9	
3	112.9	100.5	139.6	1140
	114.7	98.1	116.1	
	118.8	95.8	102.5	
	127.3	90.1	120.6	

TN-368 PLUS HS

	Peak + Background			
**Experiment #**	**Mock**	**AcWT**	**AcB2**	**Area**
1	344.7	330.3	85.2	1200
	342.4	298.0	83.6	
	330.5	297.8	85.5	
	355.7	320.3	78.9	
2	125.1	88.1	35.9	896
	102.2	80.1	27.3	
	110.3	95.4	30.6	
	111.3	90.3	26.1	
3	129.9	93.8	32.4	858
	105.2	87.1	30.5	
	113.1	98.3	30.7	
	116.1	93.9	25.4	

[1] Analysis of ^{32}P-labelled siRNAs was done for the experimental data summarized in Figures 2, 4 and 6. For data associated with Figures 4 and 6, a sub table for each set of samples is shown for each sample type (i.e. with or without HS). Each experiment (biological replicate) utilized a different, independent batch of cells, and in each experiment the dicing assay and siRNA quantification was replicated (technical replicates) two to four times.
